# Resourcefulness of propylprodigiosin isolated from *Brevundimonas olei* strain RUN-D1

**DOI:** 10.1186/s13568-023-01579-y

**Published:** 2023-07-09

**Authors:** Olumide D. Olukanni, Temitope Abiola, Jonathan B. Dada, Peter A. Dare, Femi Ayoade, Adedayo T. Olukanni

**Affiliations:** 1 Department of Biochemistry, Redeemer’s University, PMB 230 Ede, Ede, Osun Nigeria; 2 African Centre of Excellence for Water and Environmental Research (ACEWATER), Redeemer’s University, PMB 230 Ede, Ede, Osun Nigeria; 3 Department of Biological Sciences, Redeemer’s University, PMB 230 Ede, Ede, Osun Nigeria

**Keywords:** Pigment, Antibacterial, Biomarker, Textile dyes, Prodigiosin, Percentage fadedness

## Abstract

A novel red-pigmented bacterium was isolated from a water sample collected at Osun River, Ede. Morphological and 16 S rRNA gene sequencing revealed that the bacterium is a strain of *Brevundimonas olei*, while its red pigment was identified using UV-visible, FTIR and GCMS as a derivative of propylprodigiosin. The maximum absorbance of 534 nm, the FTIR’s 1344 cm^− 1^ peak of prodigiosin’s methoxyl C-O interaction, and the molecular ions from GCMS confirmed the pigment’s identity. The pigments production was temperature-sensitive (25 °C), lost at > 28 °C, and in the presence of urea and humus. In addition, the pigment turned pink in the presence of hydrocarbons, while its red colour was retained with KCN and Fe_2_SO_4,_ and enhanced by methylparaben. Furthermore, the pigment is stable in high temperature, salt, and acidic conditions, but changed to yellow in alkaline solution. The pigment, identified as propylprodigiosin (m/z 297), demonstrated broad-spectrum antibacterial activities against clinically important strains of *Staphylococcus aureus* (ATCC25923), *Pseudomonas aeruginosa* (ATCC9077), *Bacillus cereus* (ATCC10876), *Salmonella typhi* (ATCC13311), and *Escherichia coli* (DSM10974). The ethanol extract has the highest zones of inhibition of 29 ± 3.0, 26 ± 1.2, 22 ± 3.0, 22 ± 1.5, and 20 ± 2.0 mm, respectively. Furthermore, the acetone pigments interacted with cellulose and glucose such that increasing glucose concentrations showed linearity at 425 nm. Finally, the fastness of the pigments to fabrics was excellent, with percentage fadedness of 0 and − 43% light and washing tests, respectively, in the presence of Fe_2_SO_4_ as the mordant. The antibacterial nature of prodigiosin solutions and their good textile fastness to fabrics could be essential in manufacturing antiseptic materials such as bandages, hospital clothing and agricultural applications such as tubers preservation.

Key points

**Supplementary Information:**

The online version contains supplementary material available at 10.1186/s13568-023-01579-y.

## Key points

- Propylprodigiosin-producing *Brevundimonas olei* was isolated from freshwater.

b. Pigmentation was possible on agar at room temperature and induced in broth with parabens.

iii. The propylprodigiosin has potential for pharmaceutical, biotechnological and textile applications.

**Supplementary Information:**

The online version contains supplementary material available at 10.1186/s13568-023-01579-y.

## Introduction

Industrialisation is vital for national and global development, but its advancement has consequences. Some of the benefits are changes in family social status, economic growth, affordability of items and increased productivity. This drive for large-scale production earlier moved the resources for industrial use from natural to synthetic chemicals. Some of these chemicals have been proven ineffective (drug), expensive, and have serious environmental and health concerns (Affat 2021). For instance, in the pharmaceutical and textile industries, attention is being given to natural resources for drugs despite the several years of using their synthetic equivalents. Studies have shown that synthetic drugs are toxic, and some pathogens have developed resistance to them, thus rendering them less ineffective (Thaker and Wright 2015). Similarly, the toxic nature of synthetic dyes and laboratory chemicals is of great health and environmental concern.

Recently, industrial attention has been haggard back to natural dyes against their synthetic counterparts because they are considered safe, non-toxic, non-carcinogenic, and readily biodegradable (Usman et al. 2017). The major setback had been the ability to mass-produce these raw materials, as most natural pigments are from plants and animals, which will take years to mature. Using microbial products as raw material sources is now considered a viable alternative to synthetic ones, mainly because microorganisms grow faster than plants and animals. Secondly, microbes can be manipulated easily for better and enhanced product yield. In addition, product purification processes are usually easier and cheaper than plant sources. Thus microbial pigments are viable alternatives for the textile and food industries.

The recent yearning for natural pigments came from problems associated with their synthetic equivalents. As antibiotics, the resistance level of microorganisms to synthetic antibiotics increased steadily, thus posing a significant threat to human and animal health. It is generally believed that resistance could be minimised with natural drugs, of which microbial pigments are prominent (Gupta and Birdi 2017). Secondly, unlike synthetic dyes, microbial pigments are declared non-toxic or carcinogenic; thus, they can easily replace synthetic ones in the textile industries (Sen et al. 2019; Venil et al. 2020). Biologically, microbial pigments are essential in maintaining microorganisms’ physiology and molecular processes. They serve as adaptation mechanisms to various extreme environments protecting against solar radiation and other environmental factors such as low temperature and scarcity of nutrients (Celedon and Díaz 2021). They are also involved in functional processes like photosynthesis (Sutthiwong et al. 2014). These pigments’ colour formation and biological significance have important applications in industries.

The medicinal, biotechnological and industrial applications of natural pigments are gradually overshadowing their centuries-old uses in painting and decorations. They have found uses as antioxidants, antimicrobial and anti-cancer agents; they are also viable colour enhancers and additives in the food industries (Aenishanslins et al. 2016, Usman et al. 2017). Others have found applications in photo-sensitisers for dye-sensitised solar cells and textile fabric dyeing (Chadni et al. 2017; Li et al. 2017). Although various pigments have been isolated from plants, insects, ores, and microbes, microbial pigments have attracted more attention (Usman et al. 2017) because microbes grow and produce these pigments quickly and can use cheap culture media (sometimes waste materials) readily. Unlike plant and insect pigment production, climatic and weather conditions do not affect microbial pigment production. In addition, the microbes can be manipulated to produce these pigments in various colours using natural substrates (Grewal et al. 2022). These microbial pigments include carotenoids, melanins, quinones, flavins, monastics, violaceins, indigo and prodigiosin (Celedon and Diaz 2021; Sen et al. 2019).

Prodigiosin was first isolated from the bacterium *Serratia marcescens*; it is perhaps the most common microbial pigment because of its bright red colour (Woodhams et al. 2018). Although there are other red pigments of microbial origins, such as anthraquinone, monascorubramine, lycopene, astaxanthin and rubrolone, prodigiosins are much more recognised for their diverse pharmaceutical applications (Celedon and Díaz 2021). Structurally, they consist of a pyrrolyl dipyrromethene skeleton that contains a common 4-methoxy, 2–2 bipyrrole ring system. Various derivatives are also known, including the linear derivatives – prodigiosin and undecylprodigiosin and the cyclic derivatives – streptorubin B, cycloprodigiosin, and cyclononylprodigiosin (Mo et al. 2008; Darshan and Manonmani 2015). Besides the S. marcescent, prodigiosin has been reported in a few other bacteria, such as Hahella, Janthinobacterium, Pseudoalteromonas, Streptomyces, Vibrio and Zooshikella (Woodhams et al. 2018). There is, however, no report of any of the prodigiosin’s derivatives in *Brevundimonas sps.* Recently, a bright red-pigmented bacterium was isolated in our laboratory’s study on multidrug-resistant bacteria. This study thus aimed at isolating and characterising the red pigment produced by this bacterium; and investigating its bioresource potentials, particularly in the pharmaceutical, biomolecules detection, and textile dyeing application.

## Materials and methods

### Sample collection and screening for pigment production

As part of independent research on antimicrobial-resistant bacteria in River Osun, Nigeria, water samples were collected at the Ede section of the River. The water was serially diluted five-fold, and 100 µl of 10^5^ diluents was inoculated on a nutrient agar plate and incubated at 30 °C for 24 h; the organism with bright red pigment was noticed, sub-cultured and kept for further investigation.

### Identification and molecular characterisation of pigment-producing bacteria

The morphological and biochemical characteristics of the isolate were studied using Bergey’s Manual of Determinative Bacteriology. Gram’s staining reaction catalase, oxidase, and sugar fermentation tests were carried out. The 16 S rRNA gene sequencing was done at Laragen Inc., California, USA. The sequence obtained was compared to available sequences online in a GenBank database (http://www.ncbi.nlm.nih.gov) using the BLAST tool at www.ncbi.nlm.nih.gov/BLA.

### Effect of pollutants on pigment production and microbial growth

The impact of common pollutants on the red pigment production by strain D1 was determined by sub-culturing the bacterium onto nutrient agar plates containing 0.04% different pollutants: Fe_2_SO_4,_ KCN, Hydrocarbon, Humus, and Urea. The plates were incubated at 25 °C for 24 h, and bacterial growth and enhancement of pigment production were observed.

### Extraction of pigment from strain D1

Pigments were extracted from the bacteria viz.: 1 g of the red pigment on nutrient agar plates produced by the bacteria was scraped with a sterile spatula into five different glass beakers containing 5 mL solvents (Acetone, Ethanol, Ethyl acetate, Methanol or Water) with 4% HCl (Lin et al. 2019). The beakers were covered with aluminium foil and sonicated for 10 min for cell disruption to release the pigment into the solvents. Solutions were transferred into screw-cap glass tubes labelled accordingly and centrifuged for 10 min at 4000 rpm. The supernatant containing the red pigment was pipetted into sterile labelled screw-cap tubes.

### Partial characterisation of pigments

The isolated pigment was characterised using Ultraviolet-visible spectrophotometry, Fourier-transform infrared spectroscopy (FT-IR) and gas chromatography-mass spectrometry analyses. The pigments extracted with six different solvents were analysed using Pharo 300 UV-Visible spectrophotometer (Merck-millipore) in a scan mode wavelength ranging from 200 to 800 nm. The respective solvents were used as blank. The infrared spectra of the different dried extracts were done using Shimadzu FTIR-8400 S; the scanning was in the range of 4000–400 cm^− 1^ to identify the functional group present in the pigment. The Perkin—Elmer Clarus 680 system (Perkin Elmer Inc. USA) was utilised for the GCMS analysis of ethanol extract (1 ul) of the pigment with a fused silica column, packed with the elite − 5MS) capillary column (30 m in length *250 nm in diameter *0.25 nm in thickness). The carrier gas was unalloyed helium (99.99%) at a 1 ml/min constant flow rate. An electron ionisation energy method was used with 70 eV (electron Volts) high ionisation energy with 0.2 s scan time—fragments ranging from 40 to 600 m/z to detect the GCMS spectral. The injector temperature was maintained at 250 °C (constant). The column oven temperature was set at 50 °C for 3 min, raised at 10 degrees/min up to 280 °C, and the final temperature was increased to 300 °C for 10 min. The compounds present in the red pigment were identified by comparing their retention time (min), peak area, peak height, and mass spectral patterns with the spectral database of authentic compounds stored in the National Institute of Standards and Technology (NIST) library (NIST 2008).

### Stability tests of extracted prodigiosin

The stability of the extracted red pigment was monitored against salt concentration (0–2.4%), increasing temperature (25–100 °C), and pH (acid and alkaline stability). Salt and heat stability was done as described by Abdollahi et al. (2021), NaCl was added to 5 mL pigment solution at an increasing concentration of 0.6%, and the absorbance was measured at 534 nm after complete dissolution of the salt. Acid and alkaline stability tests were conducted with acetone extract of the dye, and 100 mL of the dye solution was adjusted with NaOH across the pH range (pH 3–11). Stability was measured as percentage change in colour:

$${\%}\, \text{C}\text{o}\text{l}\text{o}\text{u}\text{r}\, \text{l}\text{o}\text{s}\text{s} =\frac{{A}_{o}-{A}_{t}}{{A}_{o}}\times 100$$

### Antimicrobial activities of the red pigment

Antimicrobial activities of the pigment were determined using agar well-diffusion methods following the pattern reported by Mostafa et al. (2018) with slight modification. The bacteria used are Gram-positive bacteria: *Bacillus cereus* (ATCC10876) and *Staphylococcus aureus* (ATCC25923), and Gram-negative bacteria: *Escherichia coli* (DSM10974), *Pseudomonas aeruginosa* (ATCC9077) and *Salmonella typhi* ATCC13311). The bacterial strains were provided from the culture collection of the Environmental Biotechnology Laboratory, Department of Biochemistry, Redeemer’s University, Ede, Osun State, Nigeria. The test organisms’ 0.5 McFarland standard (1.5 × 10^8^ cells/ml) was prepared, and 100 µl was spread on Mueller Hinton agar plates with a sterile spreader for even distribution. On drying, wells were made (6 mm in diameter) in each plate using a sterile cork-borer for the five extraction solvents and labelled accordingly. The solution of pigment extract was evaporated to dryness, and (20% w/v) of dried pigment of each solvent was re-dissolved in 1% DMSO, and 100 µl of the solution was pipetted and was aseptically dispensed into each well as labelled accordingly; 100 µl of 1% DMSO was employed as a negative control. Plates were refrigerated for 30 min to allow diffusion of the solution into the agar and then incubated for 24 h at 37 °C. The antimicrobial activities of the pigment were determined by measuring the zone of inhibition with a Vernier calliper, and zones of inhibition were recorded and compared with the CLSI and EUCAST guidelines.

### Biomolecules binding with extracted red pigment

The interactions of biomolecules with extracted red pigment were investigated by adding a 1% solution of different biomolecules to acetone extract (1%) and warmed in a water bath at 40 °C for 30 min. The biomolecules used include proteins (bovine serum albumin, peptone, casein), carbohydrates (agar, glucose, cellulose), and lipids (palmitic acid and stearic acid). The spectrum of the mixture was measured at the wavelength of 200–800 nm. The procedures were repeated using extracts of other solvents. An equal volume of the extraction solvent or distilled water was used as a blank for each process.

### Evaluation of the textile colourant potential of pigment

The scope of possible applications of the red pigment produced by *Brevundimonas sp* in the textile industry was evaluated following Metwally et al. (2021) method with modifications. Chiffon, Linen, and Satin were the textile fabrics used, and they were first scoured to avoid impurities. The dyeing process used by Metwally et al. (2021) was employed for the acetone and ethanol extracts of the pigment. Each textile fabric (5 by 5 cm) was immersed in 40 ml of the solution of the respective pigment extract in a 100 mL glass beaker and incubated at 80–90 °C for 1 h. The textile fabrics were then washed twice with water. After the preliminary dyeing, some textile materials were subjected to different mordants, such as metal mordants (FeSO_4_ and CuSO_4_) and an alkali mordant (NaHCO_3_), as reported in studies (Uddin 2014; Morales-Oyervides et al. 2017; Metwally et al. 2021). Dyed fabrics were immersed in mordant (0.5 g/L), keeping the material-to-liquid ratio (MLR) at 1:20; the soaked cloth was heated at 60 °C for 20 min. The fabric samples were squeezed to remove liquid and dried overnight at room temperature.

### Colourfastness to washing and light test

Fastness was carried out upon completion of the colouring of the pigments; fabric fastness (light and wash) was carried out according to slightly modified AATCC-16 and AATCC-61 standard methods reported by Metwally et al. (2021). For light fastness, the dyed fabrics were cut into sizes of 5 × 5 cm and placed (stapled) horizontally on cardboard. The cloth materials were then exposed to sunlight for 24 h (8 am to 4 pm each day for 3 days) while the central areas were covered by black tape. For wash fastness, the dyed fabrics were washed for 30 min with a commercial detergent solution at 60 °C, the ratio of detergent to water being 1:50. The washed fabrics were then rinsed twice with water, dried, and compared to the control (same dyed fabrics that were not washed). Percentage fadedness was determined using Image J software (procedures in Additional file 1: Figs. S3, S4).

### Data analysis

The values of the antibacterial activities were recorded as mean ± SD, and values at p < 0.05 were considered significant. Percentage fadedness was determined using integrated densities obtained from NIH ImageJ, available for free download at https://imagej.nih.gov.

## Result

### Identification and characterisation of the pigment-producing bacteria

The bacterium was identified and characterised with the help of morphological characteristics and biochemical tests (Additional file 1: Table S1). The result revealed that the organism is red-pigmented, gram-negative, catalase-positive, oxidase-positive, urease positive and could ferment sucrose and maltose. In contrast, the organism cannot ferment lactose, glucose and mannitol. The organism produced the red pigments at 25 °C, not 30 °C or 37 °C.

### Molecular identification of the bacterium

**Fig. 1:**
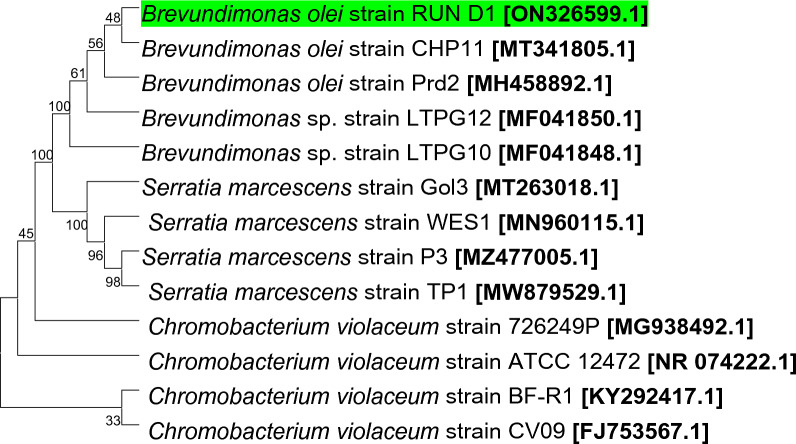
Neighbor-joining tree based on partial 16S rRNA gene sequences showing the phylogenetic position of *Brevundimonas olei* strain RUN-D1 among closely related taxa at a percentage of 1000 bootstrap replicates. Branches corresponding to partitions reproduced in less than 50% of bootstrap replicates are collapsed. The evolutionary distances were computed using the Maximum Composite Likelihood method and are in the units of the number of base substitutions per site. This analysis involved 13 nucleotide sequences

The isolate was identified molecularly using the 16 S rRNA gene sequencing and analysis as *Brevundimonas olei*. This 16 S rRNA region sequencing was exposed to a multiple alignment algorithm against the closest published sequences, and a phylogenetic tree was generated (Fig. 1) using MEGA 11 (Tamura et al. 2021). The most adjoining strain to the isolate being investigated was found to be *Brevundimonas olei*. The obtained sequence was checked for contaminants using DECIPHER (Wright et al. 2012), submitted to Genbank, and issued an accession number of ON326599. The strain was then deposited at the University of Calabar Collections of Microorganisms (UCCM) with the number UCCM00144.

### Extraction of pigment

**Fig. 2:**
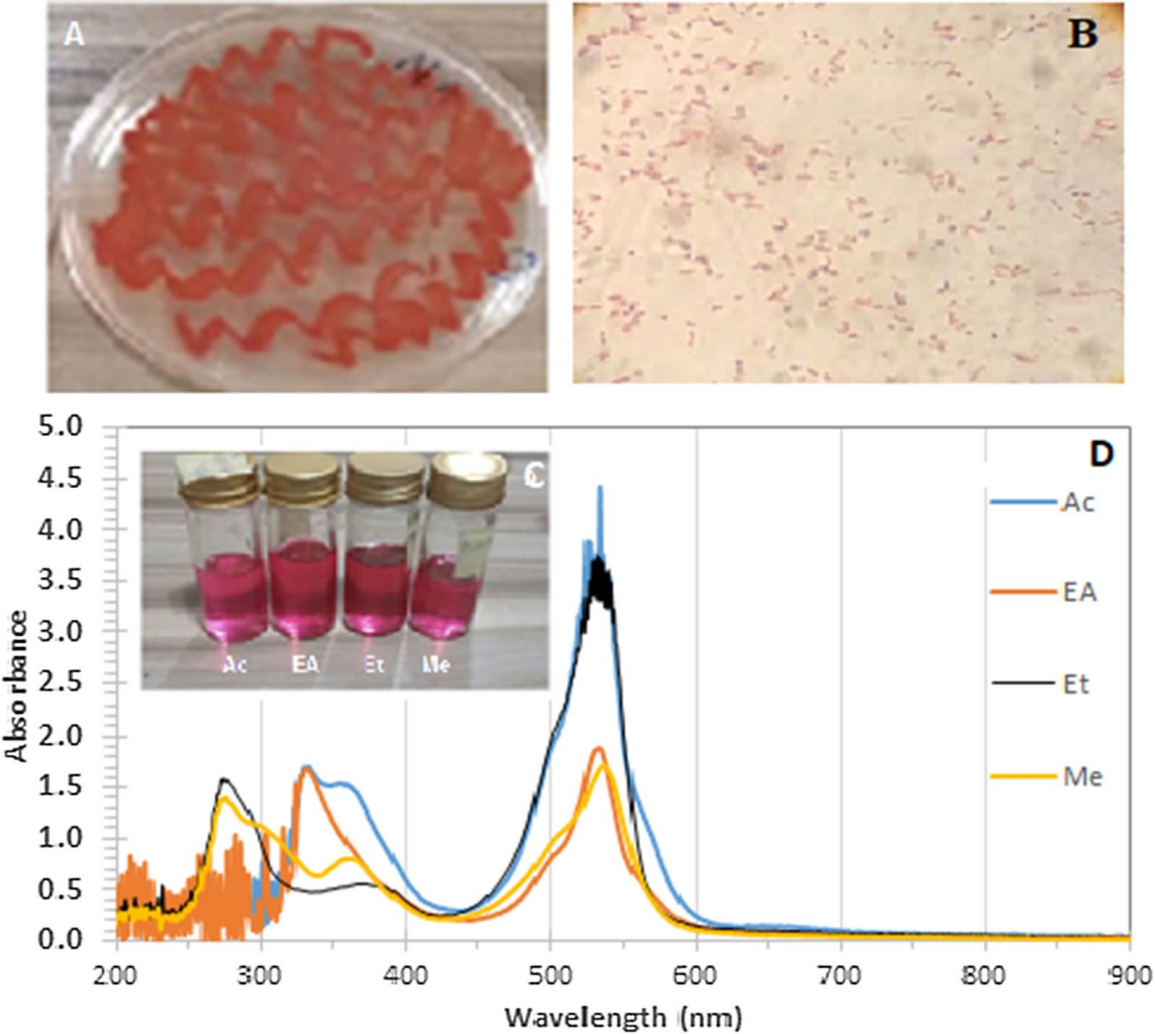
Isolated *Brevundimonas olei* (RUN-D1) on nutrient agar, **A**; Gram’s staining of pigment-producing bacterium **B**; Solutions of extracted pigments in different solvents. **C**; UV-visible spectral of the various extracts of the pigments from strain D1. **D**

**Fig. 3:**
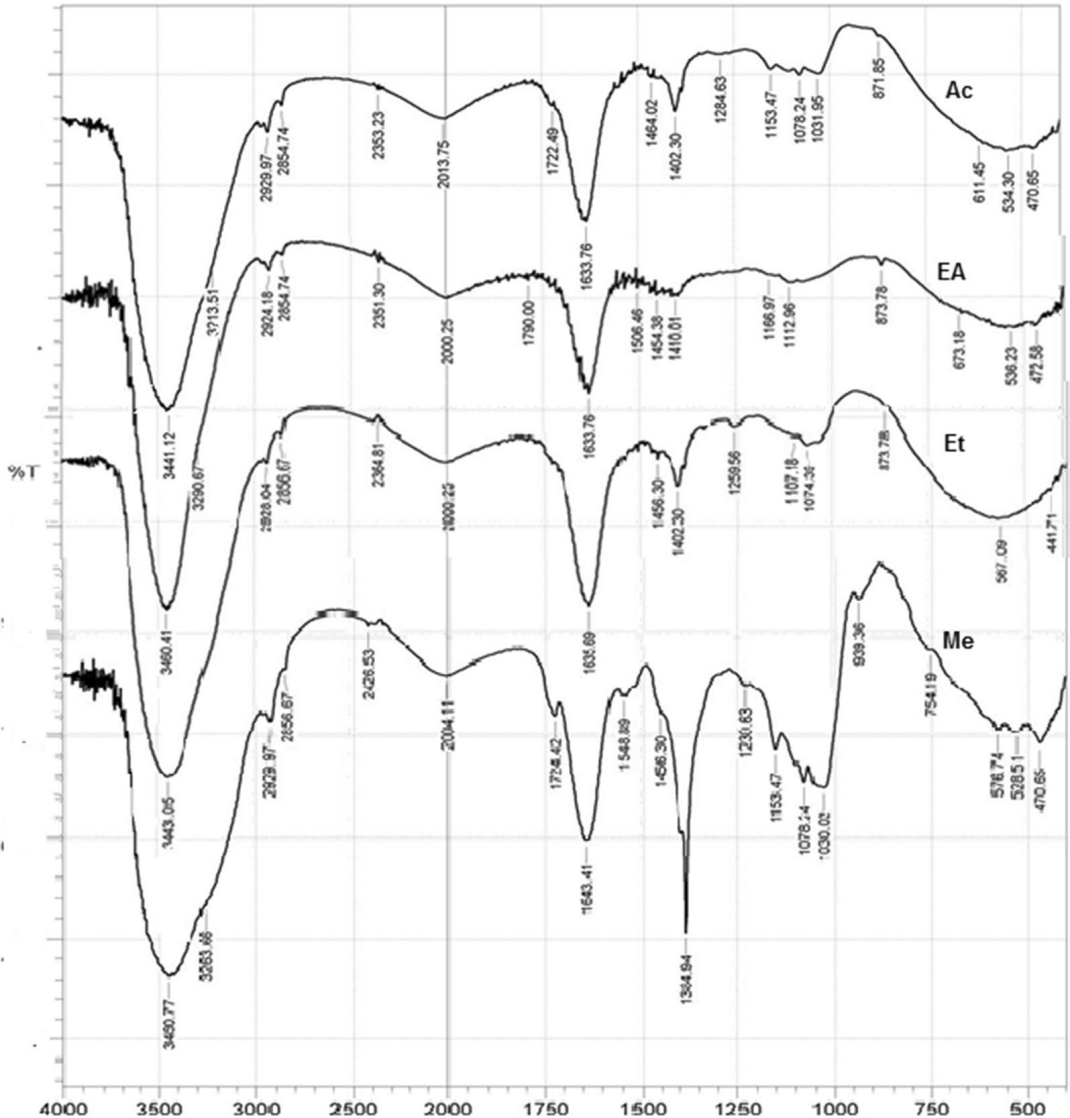
Fourier-transform infrared spectroscopy (FT-IR) of the red pigment from strain D1 extracted with acetone (Ac), Ethyl Acetate (EA), Ethanol (EtOH), and Methanol (Me)

The pigment was red (Fig. 2); varying shades obtained due to the different solvents used in the extraction were evident in the UV-visible study with diverse peaks at the UV end of the spectra (Fig. 3). Pigments extracted with water were excluded from the results because of poor colouration.

### Effect of pollutants on pigment production and microbial growth

The effect of pollutants on pigment production and microbial growth is shown in Table 1. The study showed urea and humus negatively impact pigment production by strain D1. There was, however, a change in shades from bright red to pink in the presence of diesel. The pigment production was also enhanced in the presence of methylparaben. The pollutants did not inhibit bacterial growth at the minimal concentration of 0.04% used in the study.

**Table 1:** Effect of common pollutants and temperature on microbial growth and production of red pigment in *Brevundimonas sp* (RUN-D1)

Common pollutant	Microbial growth	Nutrient agar	Nutrient broth
Control^a^ 25 °C	+	Red	Light red
30 °C	+	-	-
37 °C	+	-	-
KCN	+	Red	Light red
Fe_2_SO_4_	+	Red	Light red
Urea	+	-	-
Humus	+	-	-
Diesel	+	Pink	Light red
Methyl paraben	+	Red	Red

^a^ Bacteria culture on nutrient agar or broth without any pollutant

### Characterisation of pigment

The highest yield is with acetone, while the lowest is with methanol; from the UV-visible studies, ethanol extract seems to be the purest; thus, it was used for GCMS studies. Figure 2 showed the bacterium on a petri dish, its gram-negative nature, and the colour of the extracted pigment in different solvents; the spectrogram of the solutions showing maximum absorbance at around 534 nm was also the figure.

### Fourier-transform infrared spectroscopy (FTIR) characterisation of pigment

The FTIR spectra of the pigment produced by the *B. olei* strain D1 when extracted with various solvents were overlaid in Fig. 3. The spectra are similar in features, with two main peaks at 3460.41 cm^-1^ and 1633.76 cm^-1^; the 3460 peaks have a shoulder peak at 2929 cm^-1^. Other peaks include those at 2856.67 cm^-1^, 1635.69 cm^-1^ and 1259.56 cm^-1^. All the spectra also exhibit 1722 cm^-1^, 1633.76 cm^-1^, 1464 cm^-1^, 1402 cm^-1^, 1284.63 cm^-1^, 1078.24 cm^-1^, 1031.95 cm^-1^, and 611 cm^-1^ peaks. In addition, the spectrum of the methanol extract has a strong 1349 cm^-1^ peak; its 2460 cm^-1^, 1548 cm-1 and 1230 − 1030 cm^-1^ peaks are broader than that of the other extracts.

### GCMS result of extracted red pigment

**Fig. 4:**
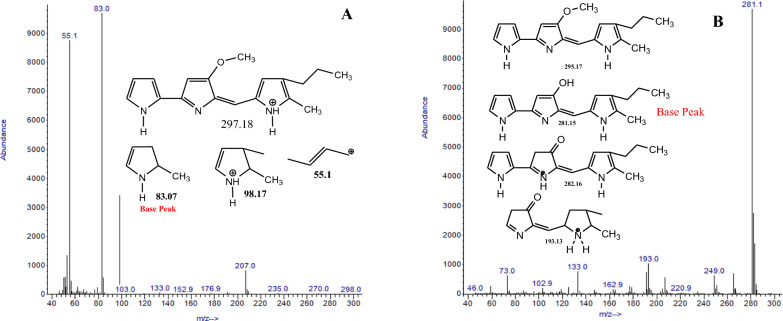
Mass spectra of the two prominent compounds identified in the GCMS analysis of the pigment showing compound 1 (**A**) and compound 10 (**B**) and their fragment ions

The gas chromatography-mass spectroscopy analysis of the red pigments revealed 85 different compounds, of which only six of them account for 78.60% of the abundance; The identity of the six prominent ones, each with a percentage abundance > 5%, was accounted for using their respective base peaks and fragmentation productions. The characteristics of the six compounds are presented in Table 2, and the mass spectra, the two most prominent ones accounting for the base peaks and other fragmentation products, are shown in Fig. 4. The detailed structures of the six major compounds are available in the supplementary file (Additional file 1: Fig. S3). The most abundant pigment was identified as 2-methyl-3-propyl-6-methoxylprodiginine (propylprodigosin) (m/z 298) at a retention time of 5.3682 min accounting for 39.50% of the extracted pigments, while the second was 2-hydroxyl-3-propyl-6-methoxylprodiginine (m/z 288) with a peak area of 17.36% (3.4681 min). The other identified compounds are prodigiosin derivatives with varied R1, R2 and R3 substituents, as shown in Fig. 4.

**Table 2:** GCMS result of prominent compounds present in the red pigment

S/N	RT	Identity	Area %	M+	Base peak
1	3.4681	Compound 1	17.3608	298.0	83
2	4.2189	Compound 5	5.7351	288.0	207
3	4.4499	Compound 6	6.3031	280.9	59
4	5.091	Compound 9	11.1520	287.0	59
5	5.3682	Compound 10	32.0517	297.0	281
6	5.5992	Compound 12	5.0030	286.0	281
**Total**			**78.6030**		

More details are available for the compounds in the additional file; the total percentage of the six prominent compounds is in bold

### Stability tests on extracted prodigiosin

**Fig. 5:**
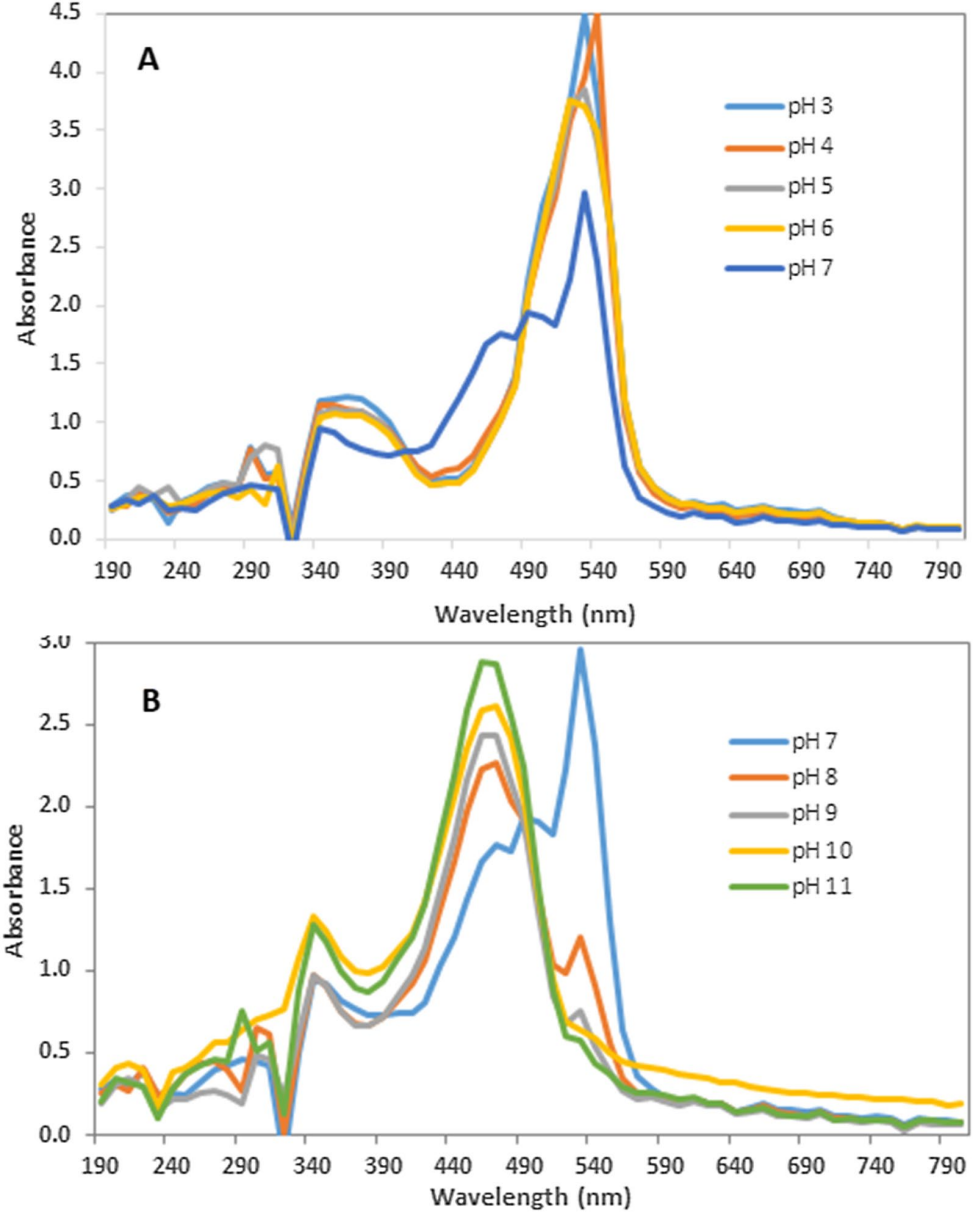
Acid (**A**) and alkaline (**B**) stability tests of propylprodigiosin extracted from *Brevundimonas olei* strain RUN-D1

The stability test results showed that both temperature and salts have no significant impact on the pigment; only 8% colour loss was experienced at 100 C. The salt stability test showed an initial colour loss of 1.19% at 1.2% salt concentration and later gained 0.5% at 2.4%. The acid and alkali stability test results are presented in Fig. 5A, B, respectively. The absorbance intensity of the pigment increased, at the pigment’s ʎ_max_, as the pH became more acidic (hyperchromic effect); however, there was no coagulation or precipitation. Precipitation was also not observed with the base stability test, but the red colour of the pigment changed to yellow, as seen in the hypsochromic wavelength shift from 534 to around 470 nm.

### Antimicrobial activities of extracted red pigment

**Fig. 6:**
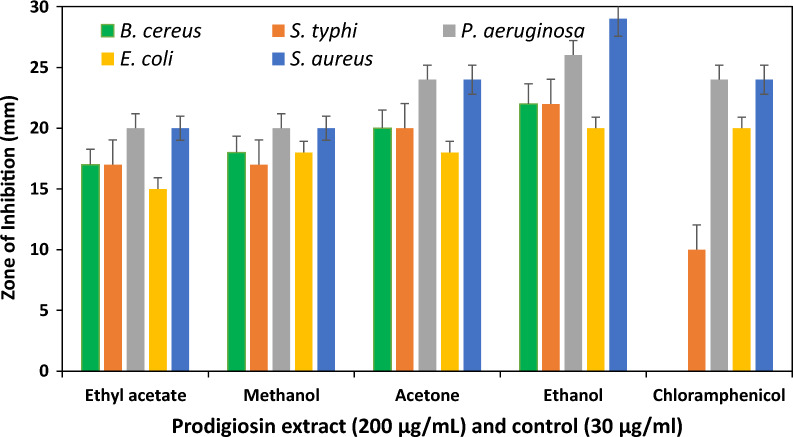
Antimicrobial activities of red pigment and chloramphenicol (control) on five renowned Gram-positive and Gram-negative bacteria

All the extracted red pigments exhibited antibacterial activity against Gram-positive bacteria such as (*Bacillus cereus* (ATCC10876) and *Staphylococcus aureus* (ATCC25923), and Gram-negative bacteria (*Escherichia coli* (DSM10974), *Pseudomonas aeruginosa* (ATCC9077) and *Salmonella typhi* ATCC13311) (Fig. 6). The ethanol extract showed the highest inhibition zones ranging from 22 ± 1.5 mm in *B. cereus* to 28 ± 3 mm in *S. aureus*. The Ethyl acetate extracts had the lowest antibacterial activities against all the bacterial strains tested, with 15 ± 1.0 mm against E coli and 20 ± 1.0 mm against *S. aureus* and *S. typhi*. The figure also showed some level of resistance of the strains to chloramphenicol, which was used as the control in the study (Fig. 6 & Additional file 1: Fig. S2).

### Interaction of pigment with biomolecules

**Fig. 7:**
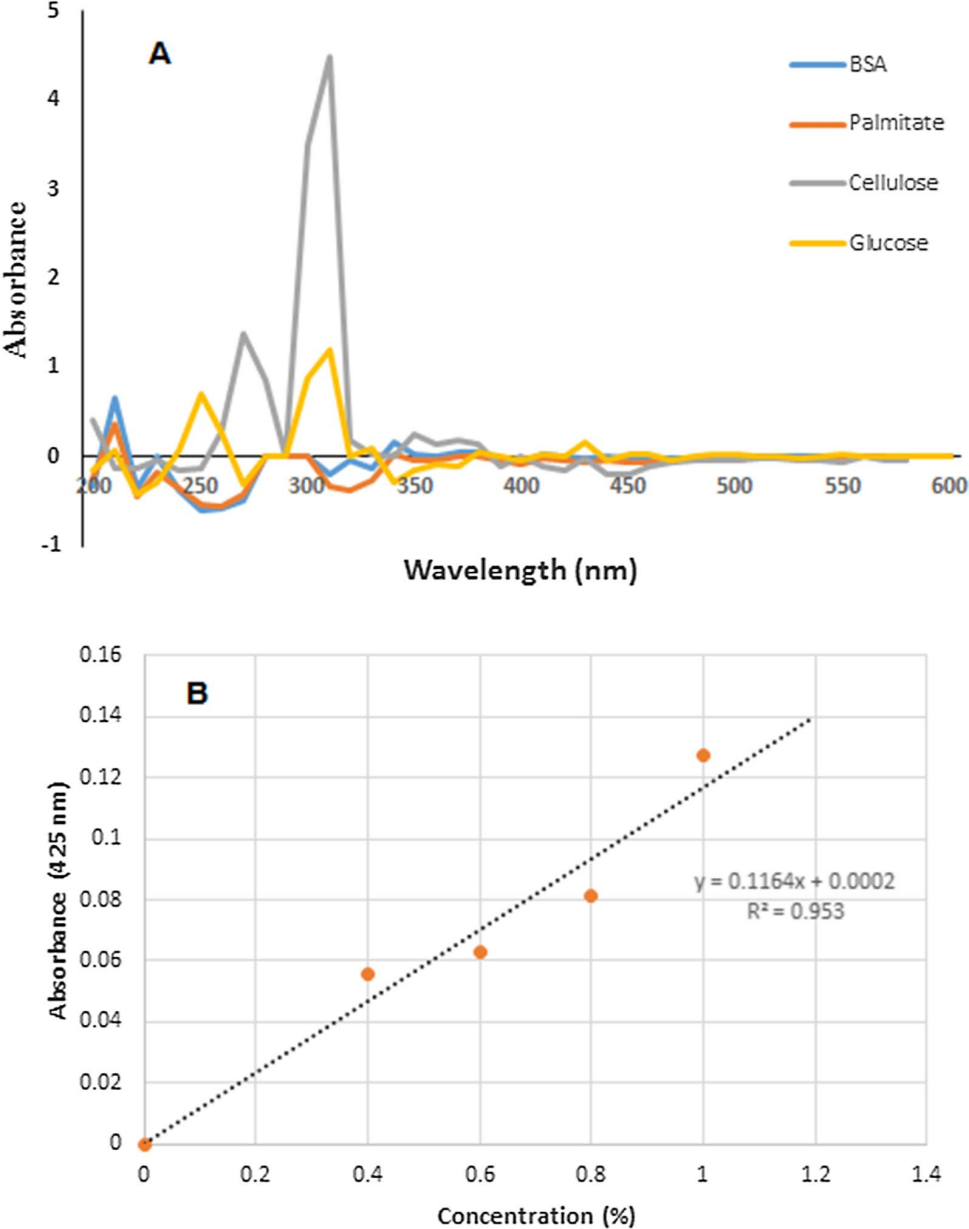
Interaction of red pigment with different biomolecules (BSA –Bovine serum albumin, palmitate, cellulose, and glucose) and quantification of glucose using the pigment

The interaction study investigated the possible detection or quantification of biomolecules using the isolated red pigment. The pigment’s ability to fluorescence in the presence or absence of selected biomolecules was monitored as part of the interaction study. Also, changes in absorbance or production of unique absorbance peaks in the presence of the biomolecules were observed using UV-visible scans. No fluorescence was observed under UV light in the presence or absence of the biomolecules in the pigment solutions. A typical result of the UV-visible scan of the interaction is presented in Fig. 6. The pigment gave different lambda max on interaction with biomolecules; the maximum absorbance was 210 nm for BSA and palmitate and 310 nm for glucose and cellulose (Fig. 7A). In the biomolecules interaction studies with the ethyl-acetate extract, there appeared to be a region of the UV (around 310 nm) specific for cellulose and glucose; a similar trend is observed for the interaction of the ethanol extract, but with cellulose alone (Additional file). Attempt to measure the different concentrations of glucose at 320 and 425 nm using its interaction with the pigment showed a linear relationship between the concentration and the absorbance around 425 nm (R2 = 0.953); the mean absorbance at 0.2% concentration was, however slightly negative (Fig. 7B).

### Evaluation of the textile colourant potential of the red pigment

**Fig. 8:**
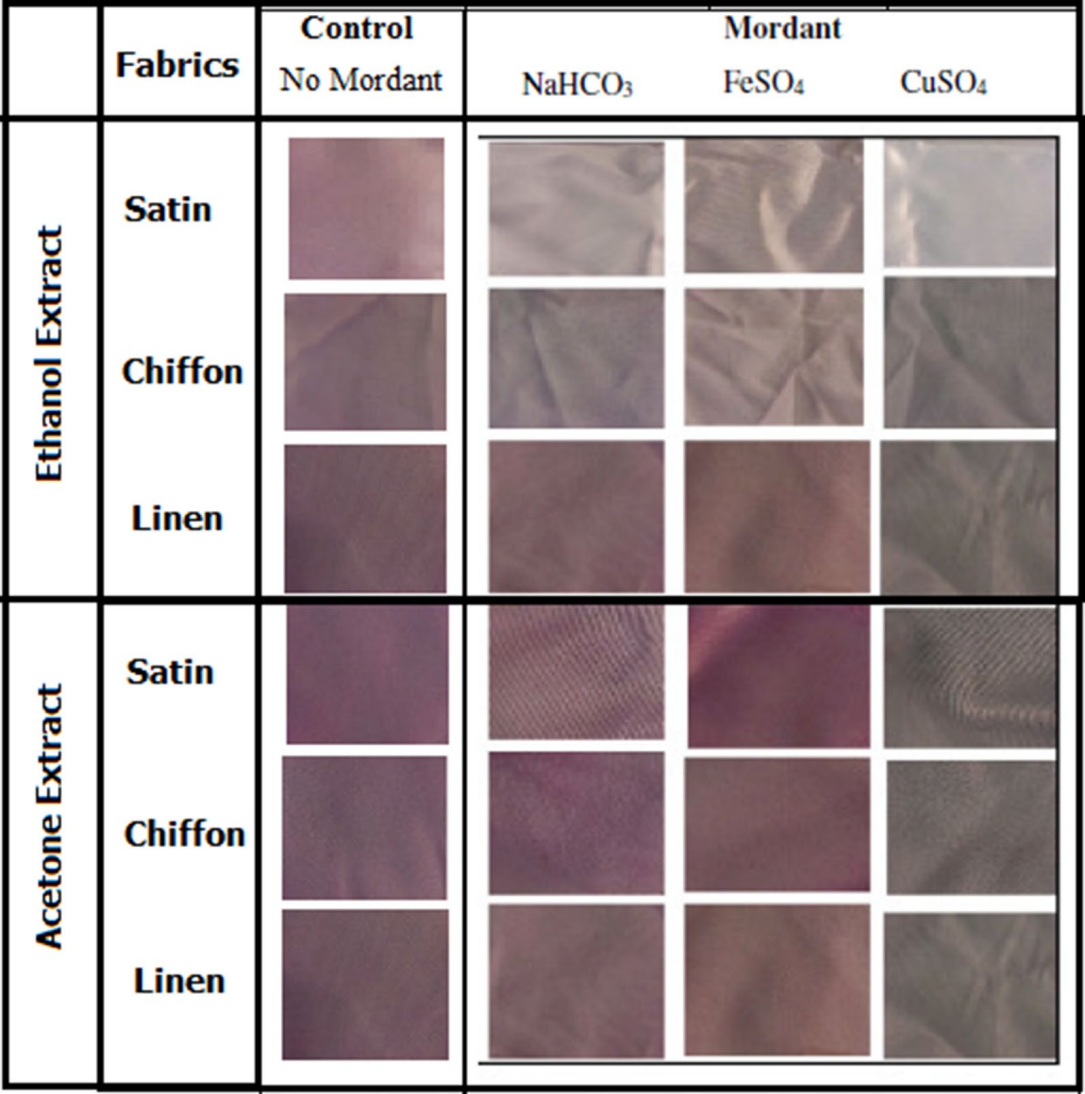
Textile fabrics dyed with ethanol and acetone extracts of the red pigment and colour hues obtained after applying different mordant to the textile materials

The experiment showed that the ethanol extract of the red pigment had a higher affinity on (chiffon, satin, and linen) textile materials than the acetone extract, as shown in (Fig. 8). The decrease in the intensity of colour was observed accordingly in this order: linen > chiffon > satin for ethanol extract of the pigment; and in the order: linen > satin > chiffon for acetone extract of the pigment. The study also showed that different mordants gave different shades of colour with the same dye, which in this case is the red pigment from *B. olei*, as shown in. When copper sulfate was used as a mordant for the acetone and ethanol extracts of the red pigment, it was observed to darken all the fabrics’ colour shades for the acetone extract. For the ethanol extract, copper sulfate brightened the shades of only the satin fabric. On the contrary, the second mordant used (iron sulphate) brightened the shades of the fabrics (linen, chiffon, and satin) for both the ethanol and acetone extracts of the pigment. The shades were more intense in satin fabric, followed by chiffon and linen for the acetone extract, while for the ethanol extract, the intensity reduced in the order of linen > chiffon > satin. Using sodium hydrogen bicarbonate as a mordant also produced bright shades of the fabrics but not as intense as that of the iron sulphate. Generally, linen and chiffon provide bright hues with the pigment’s acetone extract than with the ethanol extract of the pigment (Fig. 8).

**Fig. 9:**
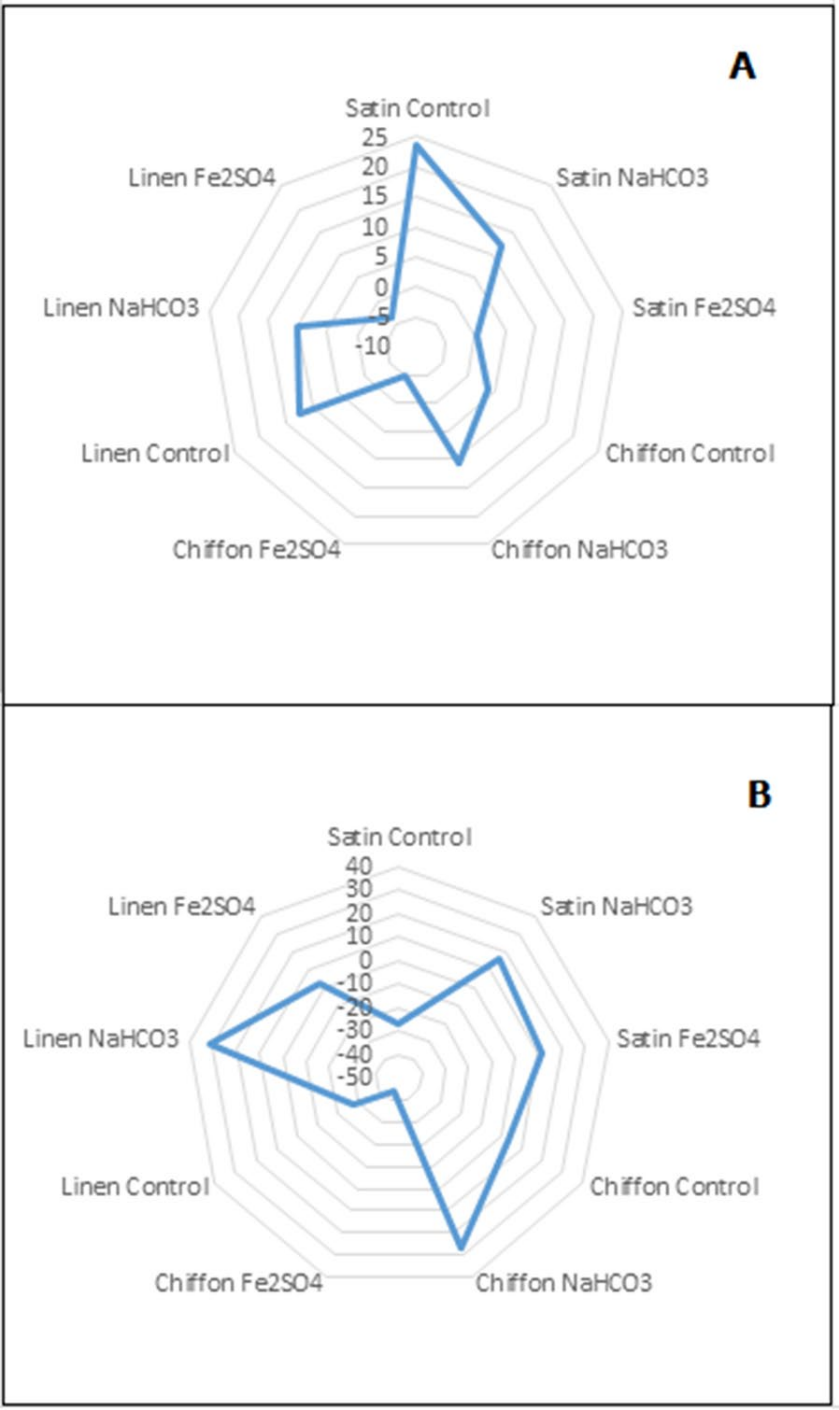
Light (**A**) and washing (**B**) Fastness tests showing the percentage fadedness of satin, chiffon and linen fabrics dyed with prodigiosin in the presence of NAHCO_3_ or Fe_2_SO_4_ as a mordant

The light-fastness and wash-fastness showed that the pigment has the most affinity to chiffon than the other two fabrics (Fig. 9). The fading of the coloured fabrics is pronounced in the control fabrics, that is, those dyed without the use of mordant; satin showed 23.53 ± 10.12% fadedness after the 24 h exposure to sunshine, while the values were 12.52 ± 8.34 and 3.95 ± 1.99% for Linen and Chiffon respectively. However, in the presence of NaHCO_3_ mordant, the % fading was reduced to 12.18 ± 3.10, 9.97 ± 0.86 and 10.62 ± 2.10%, for Satin, Linen and chiffon, respectively. However, iron sulphate showed the best mordant activities with percentage fadedness of 0.15 ± 3.29, − 5.01 ± 5.42, and − 3.57 ± 0.65% for the same set of fabrics. Unlike the light fastness, the colour intensity was generally less for all fabrics with the wash test. The fastness was 31 ± 9.22% for linen control and 2.66 ± 2.320 for Linen with Fe_2_SO4. The percentage fadedness for both light and wash fastness was determined using ImageJ (Additional file 1: Figs. S5, S6).

## Discussion

Different types of bacteria have been reported to produce various kinds of prodigiosin, and low temperatures have induced pigmentation in such bacteria. In the present study, culturing the bacterium at a temperature higher than room temperature (25 °C) resulted in growth without pigment formation. This pigmentation temperature was discovered by chance when an organism for a multidrug-resistant study was left outside the 30 °C incubator. The bacterium was subsequently identified as a strain of *Brevundimonas olei* using 16 S rRNA gene analysis.

Generally, *Brevundimonas* strains are gram-negative, motile bacteria belonging to the family Caulobacteraceae. They can be found in various environments, such as soils (Dahal and Kim 2018), water (Toth et al. 2017; Lee et al. 2020; Qu et al. 2019), and plant roots (Menendez et al. 2017). *Brevundimonas* have been utilised in soil bioremediation (Zhang et al. 2021) and in treating polluted water (Wang et al. 2015). Recently, Jiang et al. (2021) reported the isolation of a novel pyomelanin-producing *B*. *vitisensis* from a grape; however, this is the first study to report the isolation of a novel red pigment from *Brevundimonas*. Bacteria such as *Serratia marcescens, Serratia rubidaea*, *Rugamonas Rubra, Streptoverticillium rubrireticuli, Vibrio gazogenes, Alteromonas Rubra* have also been reported to produce the prodigiosin red pigment. Other bacteria like *Agrobacterium aurantiacum*, *Paracoccus carotinifaciens, and Xanthophyllomyces dendrorhous* produce a pink-red pigment known as astaxanthin (Venil et al. 2020). Islam et al. (2019) have reported a non-pigmented *B. olei* cultured at 28 ± 2 °C; pigment formation in bacteria is temperature sensitive and usually induced by one or more pollutants.

Pollutants have been known to induce pigment production in some organisms. Low temperature has been associated with the induction of pigment production in other microorganisms (Venil et al. 2020). However, the temperature varies from strain to strain and the nature of the pigments produced. Most prodigiosin-producing organisms have demonstrated that a room temperature of about 25 °C is necessary for the pigmentation and that the bacteria must be cultured on agar or selected media (Amorim et al. 2022; Venil et al. 2020). This reported observation is similar to the findings in this study; pigmentation was poor in nutrient broth but could be enhanced (in broth) in the presence of certain pollutants such as methylparaben. Faraag et al. (2017) have reported a three-fold increase in the concentration of prodigiosin produced by *S. marcescens* in the presence of L-tyrosine when compared with nutrient broth control. The non-pigmentation experienced with urea, and humus suggested that these pollutants may inhibit the transcriptional process or the enzymes responsible for prodigiosin production. It is believed that these pollutants, being good protein-denaturing substances, inactivate the enzyme(s) responsible for prodigiosin synthesis (Darshan and Manonmani 2015).

The FTIR spectrum was used to identify the functional group of the active components based on the peak value in the infrared radiation region. In the FTIR analysis, samples are bombarded with infrared (IR) radiation. The IR radiations then allow for the vibrations of the atoms in a molecule of the sample, which leads to the precise absorption and/or transmission of energy (Nandiyanto et al. 2019). The broad absorption between 3600 and 3000 cm^− 1^ and peaks at 3334 cm^− 1^ corresponds to N–H stretching; the high intensity is ascribed to the presence of the moiety responsible for this peak (Faraag et al. 2017). The spikes at 2979 and 2828 cm^− 1^ were attributed to C–H stretching and asymmetric stretching of CH_2_, respectively. The peak at 1722.49 cm^− 1^ is assigned to ketones which form bands at 1725−1705 cm^− 1^, while the peak formed at 1633.76 cm^− 1^ was due to Alkenyl C=C stretch (Aljani et al. 2022, Stoyanov et al. 2021). Prodigiosin exhibits very strong bands at 1633 due to the presence of –NH. The 1153 and 1107 cm^− 1^ peaks were attributed to C–O stretching (Faraag et al. 2017). The peaks around 1217 cm^− 1^ and 762 cm^− 1^ are attributed to the carbon-carbon double bond of prodigiosin. The characteristic peak of a pyrrole group of prodigiosin is said to be between 1369 cm^− 1^ and 1341 cm^− 1^ (Amorim et al. 2022; Sumathi et al. 2014), a prominent 1349 cm^− 1^ peak corresponding to the presence of C–O group in prodigiosin was found in the spectrum of the methanol extracts. This peak suggested that the ester bond of the methoxyl substituent existed mainly as hydroxyl in the extracts of other solvents and that the former predominated in the methanol extract of the pigments. Hydroxyl or amide group could be accounted for by the broad nature of the 2900–3600 cm^− 1^. Essentially, the group accounts for the amide and hydroxyl groups in the pigments.

Gas chromatography-mass spectrometry (GCMS) is a versatile molecular ion and structure elucidation tool. It identifies different substances within a test sample and explicitly identifies the compounds present in the substance (Kell et al. 2005). The molecular masses of the pigments identified from *B. olei* were less than 500 m/z, typical of prodigiosin (Anwar et al. 2020). However, it differs from the reported ones in *S marcescens*, which usually have a molecular weight 320 (dos Santos et al. 2021). The molecular weight of the one under study is 297 suggesting a propylprodigiosin as against the commonly reported pentylprodigiosin. Propylprodigiosin-producing bacteria have not been reported to the best of our knowledge, and we are yet to lay our hands on a report on prodigiosin-producing *Brevidomonas olei*. However, literature has suggested different derivatives of prodigiosin such as prodigiosin (2-methyl-3-pentyl-6-methoxyprodiginine), undecylprodigiosin metacycloprodigiosin, streptorubin B, and cycloprodigiosin (Bikash et al. 2021; Habash et al. 2020).

The stability of the pigment at high temperatures and high salt concentrations suggested its application in industrial processes where such temperature and salts are required, particularly the polythene and textile dyeing processes. Abdollahi et al. (2021) reported similar heat and stable microbial pigment. The stability of the pigments in the acidic solution is commendable; Product information on prodigiosin from S marcescens showed that the pigment is stable in an acidic medium and unstable in a basic medium. The stability may be attributed to the conversion of the protonated form of the pigment to a more stable reduced state by the H + made available by the acid. On the contrary, the basic medium will cause the pigment to deprotonate, distorting the conjugated bonds in the pigment rings, resulting in altered colour. The hypsochromic shift resulted in the pigment colour change to yellow similar to the one reported when toluene: ethylacetate (4:1) was used for prodigiosin elution (El-Bialy et al., 2015). The acid stability suggested that the dyeing pH will greatly contribute to the pigment’s fastness, and the altered colour in alkaline conditions indicated the possibility of using the pigment in acid-base titration classes.

Antimicrobial activities of prodigiosin are well reported (Danevčič et al. 2016; Lapenda et al. 2015), and only a few recent literatures examine the mechanism of its actions (Yip et al. 2021). In this study, we investigated the antibacterial activities of prodigiosin extracted with various solvents. This in vitro antibacterial activity assay showed that all extracts have antibacterial activities. However, the highest was reported in the pigments extracted with ethanol; this suggested that various solvents extracted a particular derivative of the prodigiosin more than the others and that the potency of the pigment is structure-dependent. It was observed through the GCMS study that the prominent compounds in the pigments are methoxyl and hydroxyl derivatives of propylprodigiosin, and one would expect anyone that predominates in the solvent used to affect the antibacterial activity. The study suggested that the solvent used for extracting the prodigiosin may have a beneficiary effect on the bioactivities of the pigment.

The results of the biomolecules’ interaction with the pigments extracted with different solvents showed that acetone and ethanol appeared to purify a more specific derivative of the pigments. At the same time, methanol and ethyl acetate extracts are a mixture of prodigiosin derivatives. In the absence of biomolecules, all extracts have a lambda max of 534 nm, typical of prodigiosin. The UV peak at 310 nm, specific for glucose and cellulose, suggested the interaction of the pigment with carbohydrates. Thus the possibility of detecting the same at the wavelength in the presence of prodigiosin. The interactions with cellulose supported the prospect of the pigment for textile dyeing. The linear relationship between the concentration of glucose and the absorbance at 425 nm showed that the biomolecule could be quantified using this pigment. However, further studies are needed to establish more suitable conditions and detection limits more that the absorbance value of the 0.2% glucose concentration was negative. This study is thus reporting, for the first time, the possibility of using prodigiosin as a biomarker for biomolecules detection and quantification.

The chemical composition of textile fabric materials is essential in dyeing because it influences the binding of the dye to the functional group(s) on it. The result obtained is due to the inability of the dye molecule to form a hydrogen bond with the hydroxyl group of the cellulose molecule, as previously reported by Ahmad et al. (2012) and Metwally et al. (2021). Mordanting is a crucial phase in the dyeing fixation process; it requires metal ions to form an insoluble precipitate on the fibre surface to achieve better dye fixation of natural dyes. A mordant is an agent added to a textile substrate to alter the dye-fibre interaction to provide better absorption, fastness, and/or a colour shade change (Metwally et al. 2021). The mordant used in this study gave various shades to the red pigment, forming some new dull colours. Various forms of mordants with a specific natural dye may brighten, darken, or significantly transform the dye colour resulting in new colour shades of the final output, giving a positive effect or an unwanted phenomenon (Uddin 2014).

Light exposure and washing are significant phenomena responsible for fabrics’ fading within the textile industry. In the presence of visible and ultraviolet light and other factors like humidity and oxygen, fading readily occurs (Forster et al. 2017). In this study, portions of the dyed fabrics (with or without mordant) were masked and exposed to sunlight for 24 h (Additional file 1: Fig. S3). The experimental outcomes showed that the newly isolated prodigiosin exhibited excellent light-fastness properties for the fabrics used: satin, chiffon and linen. The reduced fading in the presence of mordant showed that the pigment would be better as a textile pigment in the presence of suitable mordant such as Fe_2_SO_4_. The excellent fastness buttresses previous ideas that microbial pigments could be viable alternatives to synthetic dyes in textile applications (Ahmad et al. 2012; Metwally et al. 2021). The prodigiosin-dyed fabrics showed good colourfastness towards washing, as shown in the pictures (Additional file 1: Fig. S3) when the washed samples were compared to the unwashed samples. Linen with Iron sulphate showed the closest value to 0% fadedness, which is expected since the colour of the dyed linen was not as bright as other fabrics. A negative % fadedness obtained in this wash test indicates the deviation of colour from the original colour. The tendency of dye washing out on laundering is affected by many factors (Ahmad et al. 2012; Metwally et al. 2021). A grayscale of 3 and 3–4, corresponding to 40 and 30% fadedness, is acceptable in the industry for both the light and wash fastness. None of the fabrics dyed showed more than 44% loss of colour, confirming the suitability of the pigment for industrial textile application.

In conclusion, the results obtained from the various biochemical, morphological and phylogenetic analyses signify that strain D1 represents a novel member of the genus *Brevundimonas*. The phylogenetic trees obtained using the 16 S rRNA showed the associations of the strain with other strains of the *Brevundimonas* genus and that it is closely related to *olei* species. Like most prodigiosin reported, the pigment’s antibacterial activities were apparent. It also showed the potential to be used in biomolecule detection and quantification. Its fabrics’ fastness also suggested great potential in the textile industries. Efforts are being made to purify further and characterise the mix-pigments using preparative HPLC and NMR. While the former is yet to be installed, the latter is unavailable in our laboratory. *Brevundimonas olei* producing propylprodigiosin with potential in pharmaceutical, textile industries, and biomolecule identification, has been isolated from a water sample from Osun River, Nigeria.

## Supplementary information

**Additional file 1: Table S1.** Morphological and Biochemical Characterization of the Pigment Producing Microorganism. **Figure S2.** Mass Spectra of the six prominent compounds, which turned out to be derivatives of propylprodigiosins. **Figure S3.** The detailed structures of the six prominent compounds identified asderivatives of prodigiosin. **Figure S4.** Antimicrobial activities of ethanol extracts of prodigiosin and chloramphenicol (control). **Figure S5.** The UV-visible spectra of various solvent extract showing possible interaction withbiomolecules (BSA, Cel-cellulose, Glu – glucose, Pal – palmitate). **Figure S6. **Light fastness test of prodigiosin dyed fabrics, percentage fadedness determined using imageJ software. **Figure S7.** Wash fastness test of prodigiosindyed fabrics, percentage fadedness determined using imageJ software.

## Data Availability

The datasets generated for this study are available in the NCBI database. The accession number for 16 S rRNA of *Brevundimonas olei* strain RUN-D1 is ON326599. Other tables and figures are available in the Additional file.
