# Supplementary material for: Resourcefulness of propylprodigiosin isolated from Brevundimonas olei strain RUN-D1
Source: AMB Express. 2023 Jul 9;13:71. doi: 10.1186/s13568-023-01579-y (PMC10329970; doi:10.1186/s13568-023-01579-y)
Supplement: Supplementary file 1 — Additional file 1: Table S1. Morphological and Biochemical Characterization of the Pigment Producing Microorganism. Figure S2. Mass Spectra of the six prominent compounds, which turned out to be derivatives of propylprodigiosins. Figure S3. The detailed structures of the six prominent compounds identified asderivatives of prodigiosin. Figure S4. Antimicrobial activities of ethanol extracts of prodigiosin and chloramphenicol (control). Figure S5. The UV-visible spectra of various solvent extract showing possible interaction withbiomolecules (BSA, Cel-cellulose, Glu – glucose, Pal – palmitate). Figure S6. Light fastness test of prodigiosin dyed fabrics, percentage fadedness determined using imageJ software. Figure S7. Wash fastness test of prodigiosindyed fabrics, percentage fadedness determined using imageJ software. [file 13568_2023_1579_MOESM1_ESM.docx]

**Additional file**

**AMB Express**

**Resourcefulness of propylprodigiosin isolated from *Brevundimonas olei* Strain RUN-D1**

**Olumide D. Olukanni^1,2^, Temitope Abiola^1,2^, Jonathan B. Dada^1^, Peter A. Dare^3,2^, Femi Ayoade^3^, Adedayo T. Olukanni^1^**

^1^Department of Biochemistry, Redeemer’s University, PMB 230 Ede, Ede, Osun State, Nigeria

^2^African Centre of Excellence for Water and Environmental Research (ACEWATER), Redeemer’s University, PMB 230 Ede, Ede, Osun State, Nigeria

^3^ Department of Biological Sciences, Redeemer’s University, PMB 230 Ede, Ede, Osun State, Nigeria

Olumide D. Olukanni, [0000-0003-1830-9412](https://orcid.org/0000-0003-1830-9412)

Temitope Abiola, **0000-0001-6344-3958**

Jonathan B. Dada, [0000-0003-1834-6256](https://orcid.org/0000-0003-1834-6256)

Peter A. Dare, [0000-0002-4795-3090](https://orcid.org/0000-0002-4795-3090)

Femi Ayoade, **0000-0002-6599-3109**

Adedayo T. Olukanni [0000-0003-2059-7273](https://orcid.org/0000-0003-2059-7273)

Corresponding author: Olumide D. Olukanni, [olukannio@run.edu.ng](mailto:olukannio@run.edu.ng)

**Morphological and Biochemical Characterization**

The morphological and Biochemical characterization of the bacterium is presented in the table below. Generally, *Breviundimonas* are gram negative rods, non-fermenter of glucose and the catalase and oxidase activities seems to vary within species.

**Table S1: Morphological and Biochemical Characterization of the Pigment Producing Microorganism**

| Red pigmented bacteria | Result |
| --- | --- |
| Gram stain | -ve |
| Shape | rod |
| Catalase test | **+** |
| Oxidase test | **+** |
| Urease test | **+** |
| Glucose fermentation | **-** |
| Maltose fermentation | **+** |
| Lactose fermentation | **-** |
| Mannitol fermentation | **-** |

**Estimation of yield**

Yields were calculated from the absorbance of the extracted pigment in the respective solvents using the formula:

$$TPG ({mg}/L)= \frac{A\times V\times{10}^{4}}{E_{1cm}^{1\%} \times M}$$

Where A is the absorbance at 535 nm, Volume of solvent used, E extinction coefficient of prodigiosin, 0.4311 Lmg^-1^cm^-1^ (Elahian et al. 2013), and M is mass of pigment used in mg. Different values of E (extinction coefficient) have been used in different studies, however, 0.4311 Lmg-1cm-1) was chosen in this study due to the fact that it is very close to thevalue obtained from another study where the extinction coefficient was confirmed through quantitative 1H-NMR (Domrose et al. 2015).

**Identification using Gas Chromatography coupled with Mass Spectrometer**

The GCMS chromatogram for the pigments extracted with ethanol is shown in figure S1. Acidified ethanol is commonly used for prodigiosin extraction from the bacteria host and culture because prodigiosin decomposes fast above pH 5. Most of the compounds have retention time between 3.8 and 8 minutes, suggesting that they are similar in structure. The similarity in structure was confirmed by the closeness of the m/z values of the compounds molecular ions
(figure S2).


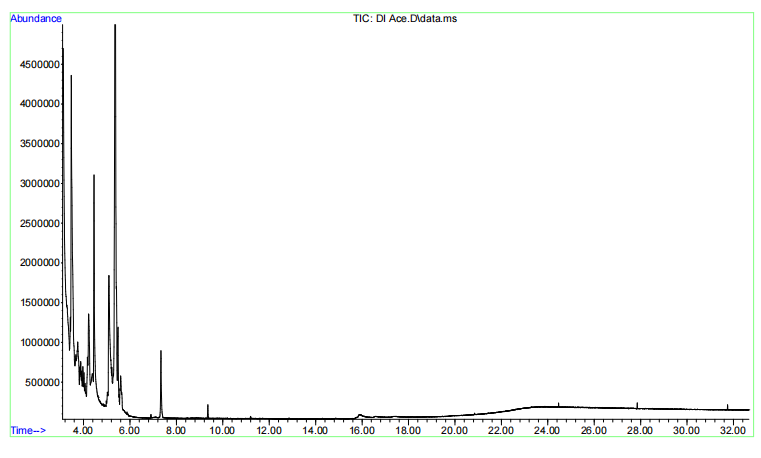
Figure S1**:** Gas Chromatogram of the GC-MS result of ethanol extracted red pigment from strain RUN-D1

**
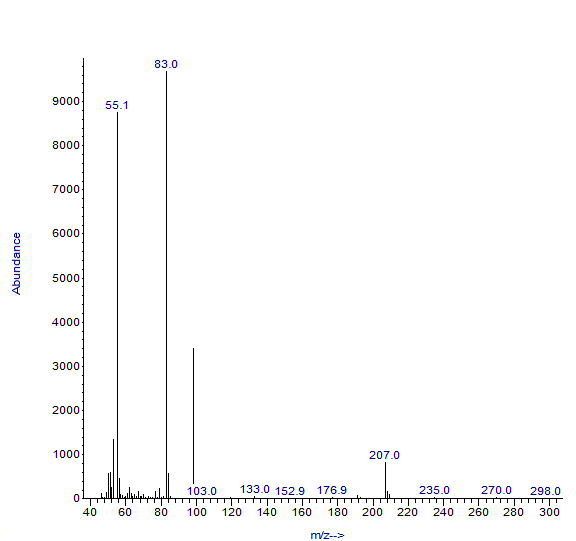

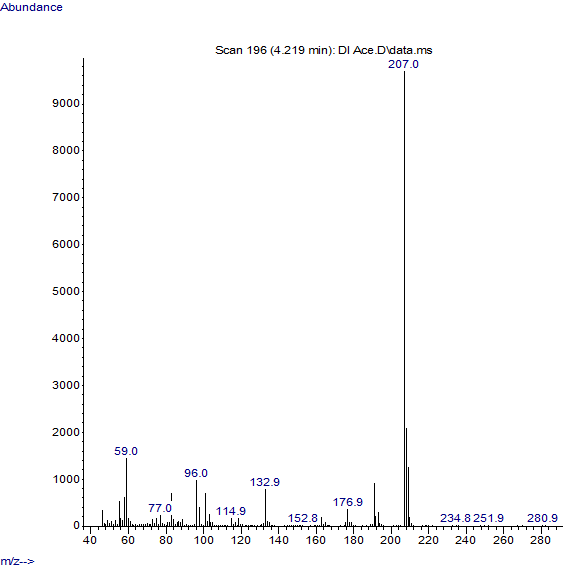
**

**
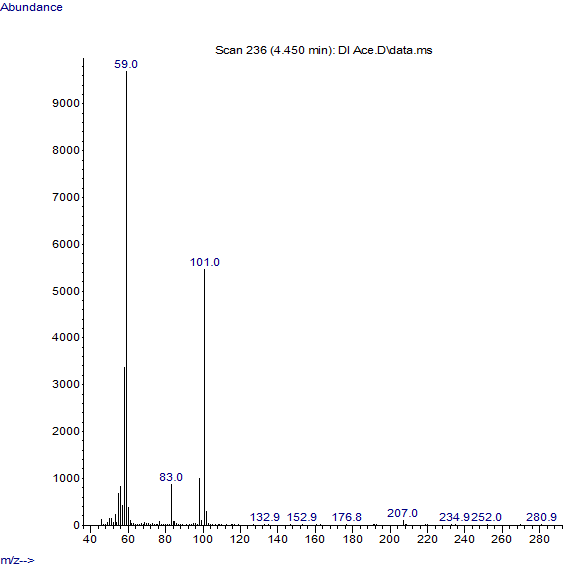

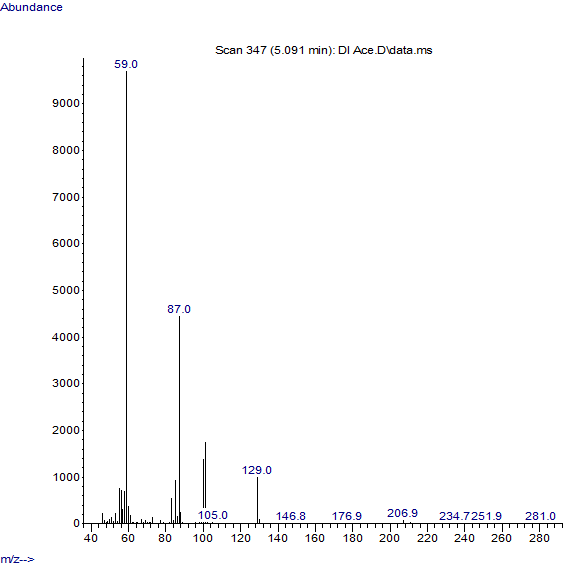
**

**
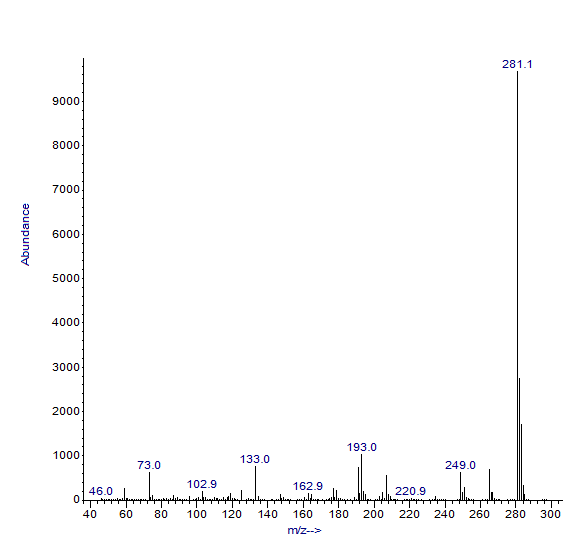

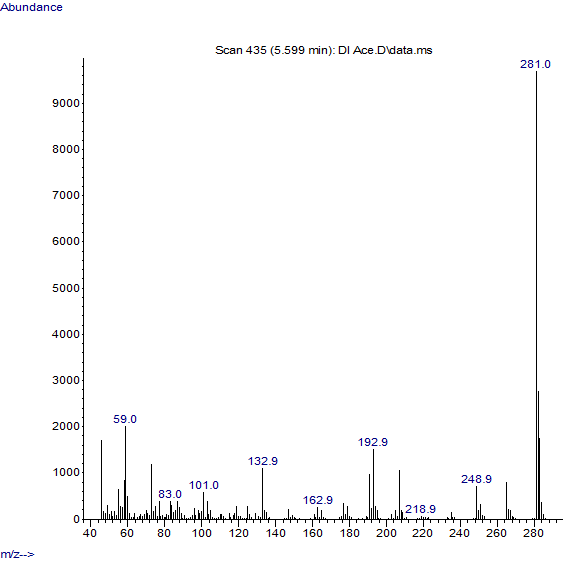
**

Figure S2: Mass Spectra of the six prominent compounds, which turned out to be derivatives of propylprodigiosins

Using the m/z values of the base peaks and the molecular ions of the various compounds, the structures of the compounds were determined and confirmed with other fragments on their mass spectra (Figure S3).

Figure S3: The detailed structures of the six prominent compounds identified as derivatives of prodigiosin

Wang et al. (2020) has identified various prodigiosin derivatives reported in literature as PGs include prodigiosin (PG, 2-methyl-3-pentyl-6-methoxyprodiginine), undecylprodigiosin, metacycloprodigiosin, streptorubin B, and cycloprodigiosin.

**Antibacterial activities of extracted pigment**

Antimicrobial activities of various derivatives of prodigiosin have been well documented, it is therefore important to verify the antibacterial activities of this newly reported derivatives. The prodigiosin was applied at 200 ug/mL concentration while the control antibiotics disc was used as supplied (30 ug/mL). The result showed that the pigment has antibacterial activities against all bacterial tested, even against those that are resistant to the control drug (Figure S4).


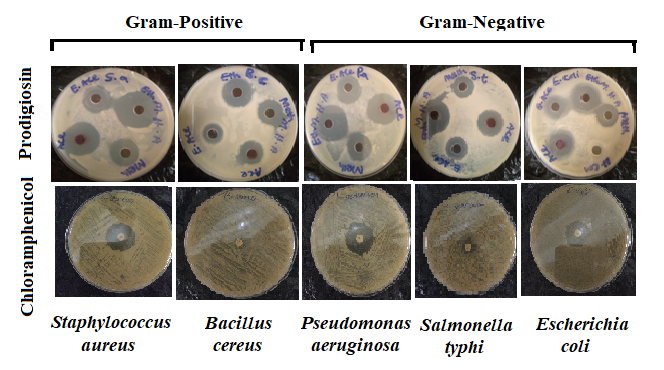


Figure S4: Antimicrobial activities of ethanol extracts of prodigiosin and chloramphenicol (control)

**Interactions of Biomolecules with Prodigiosin**

The pigment gave different absorbance maximum (visible region) values in different solvents 542 nm in acetone, 538 nm in ethyl-acetate, 537 nm in ethanol, 543 nm in methanol in the presence of the biomolecules introduced. In the presence of acetone, all the biomolecules gave a depression in lieu of peaks at 310 nm (Figure S5). The interaction of the acetone extract with the biomolecules seems to provide peaks around 275-288 nm that are unique to BSA.

Figure S5: The UV-visible spectra of various solvent extract showing possible interaction with biomolecules (BSA, Cel-cellulose, Glu – glucose, Pal – palmitate)

In the biomolecules interaction studies with the ethyl-acetate extract, there appeared to be a region of the UV (around 250 nm) that is specific for cellulose and glucose, a similar trend is observed for the interaction of the ethanol extract with cellulose alone. The peaks in the biomolecules interactions with methanol extract were diverse in nature but the BSA seemed to produce a major peak at 284 nm, while the cellulose demonstrated several interactions in both the visible and UV region, those in the UV appeared to be unique in a way.

The results of the biomolecules interaction with the pigments extracted with different solvents showed that Acetone and ethanol appeared to purify a more specific derivatives of the pigments, while that of the Me and EA are mixture of the of the prodigiosin derivatives. In the absence of the pigment, all the extracts have lambda max of about 534 nm. The 275-288 nm in the interaction of the acetone pigments with BSA suggested that the pigment could be used to distinguish BSA or proteins in the presence of other biomolecules. This showed that prodigiosin is able to bind and interact strongly with the aromatic amino acids in the bovine serum albumin, with the interactions stemming from the oxygen groups in prodigiosin interacting with the oxygen or nitrogen groups in the residues of amino acids in BSA. Other studies have also confirmed the interaction of prodigiosin with both bovine serum albumin and even human serum albumin (Rastegari et al. 2016; Liu et al. 2012). This property of prodigiosin can therefore be employed in the detection and even characterization of proteins.

**Procedure for the Light fastness test**

Different fabric, Satin, Chiffon and Linen dyed with acetone extract of the prodigiosin were rinsed and dried. Black tape was placed across each of the fabrics and the cloths were exposed to sunlight for 24 h, 8 hours each day for 3 days. The Percentage fadedness was determined as

$$\frac{({ID}_{W}-{ID}_{B})-({ID}_{W}-{ID}_{E})}{({ID}_{W}-{ID}_{B})} \times100$$

Where ID_W_, ID_B_, and ID_E_ are integrated density of white background, blocked portion and exposed portion of the dyed fabrics respectively. Using ImageJ, squares of equal areas were drawn on both the blocked portion and exposed portion in triplicates (figure S6), and the integrated density were copied from the analysis table into Ms Excel sheet for calculation.

Acetone extract of prodigiosin was used for this test rather than the ethanol extract because even though prodigiosin extracted with ethanol is stable under natural light indoor, it gets decomposed easily when exposed to sunlight. Prodigiosin is a non-toxic pigment that can be used as a dye for different fabrics including natural and synthetic. Man-made fabrics dyed with prodigiosin have been reported to show high colorfastness to washing and to also demonstrate antimicrobial activity against *E.coli* and *Staphylococcus aureus* (Liu et al. 2013).


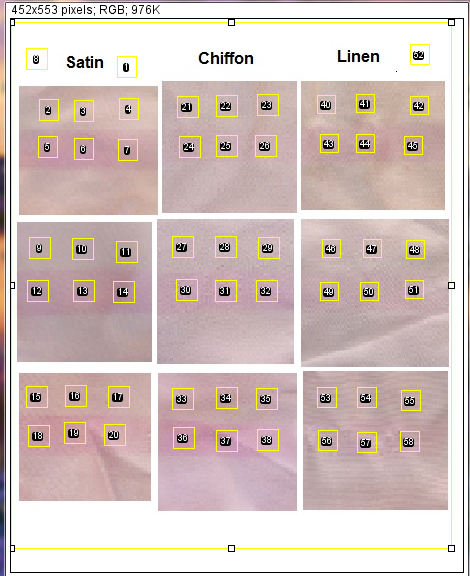


Figure S6: Light fastness test of prodigiosin dyed fabrics, percentage fadedness determined using imageJ software

**Procedure for the Wash fastness test**

Figure S7 shows the procedure for the wash fastness test showing the dyed fabrics Before washing (left) and after washing (right)

Different fabric, Satin, Chiffon and Linen dyed with acetone extract of the prodigiosin were rinsed and dried. The fabrics were hand washed in the presence of SDS as detergent. Percentage fadedness was determined in image using the with background as reference.

Percentage fadedness was determined as

$$\frac{({ID}_{W}-{ID}_{FB})-({ID}_{W}-{ID}_{FA})}{({ID}_{W}-{ID}_{FB})} \times100$$

Where ID_W_, ID_FB_, and ID_FA_ are integrated density of white background, dyed fabrics before washing and dyed fabrics after washing respectively. This formula can only be used if the area of the portion selected is the same throughout the density measurement, otherwise the percentage should be calculated individually and the percentage after washing is taking away from that before washing.


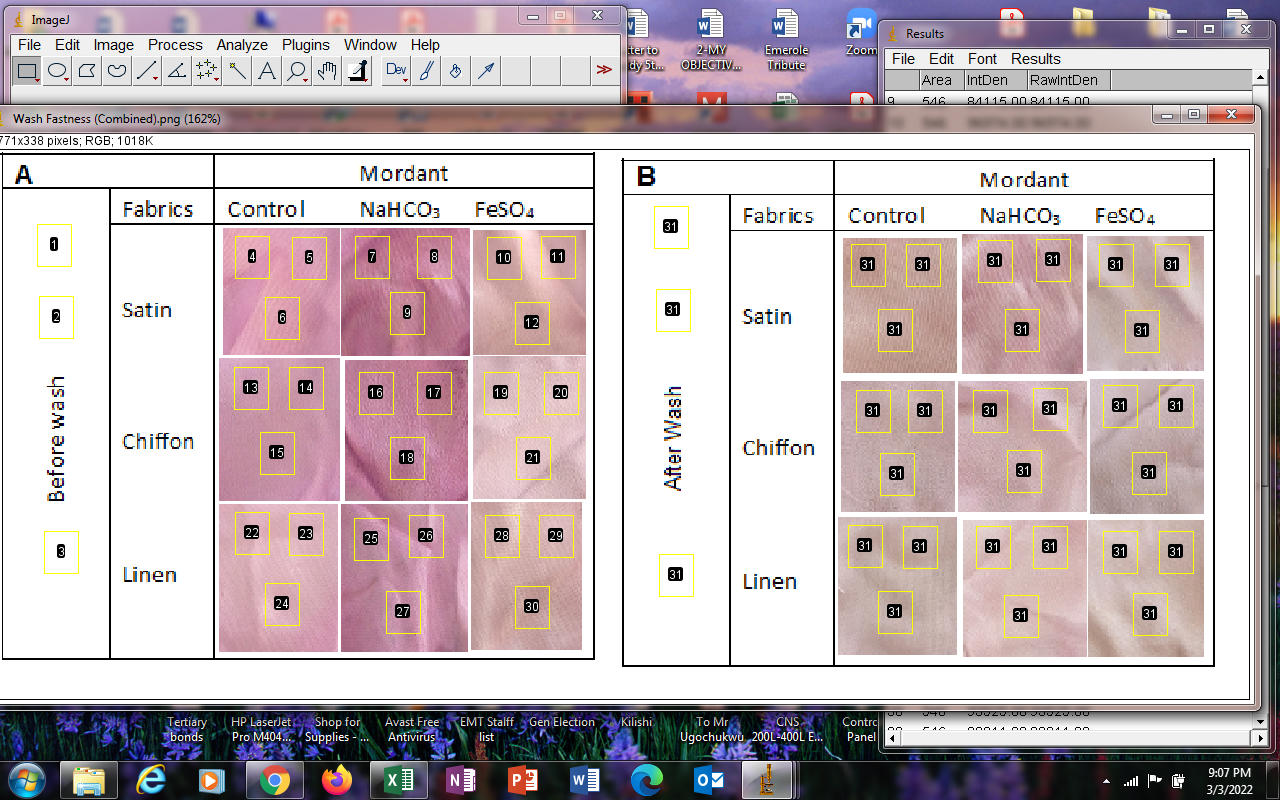


Figure S7: Wash fastness test of prodigiosin dyed fabrics, percentage fadedness determined using imageJ software.

**References**

Domrose A, Klein AS, Hage-Hulsmann J, Thies S, Svensson V, Classen T, Pietruszka J, Jaeger KE, Drepper T, Loeschcke A (2015) Efficient recombinant production of prodigiosin in *Pseudomonas putida*. Front Microbiol 6: 972.

Elahian F, Moghimi B, Dinmohammadi F, Ghamghami M, Hamidi M, Mirzaei SA (2013) The Anticancer Agent Prodigiosin is not a Multidrug Resistance Protein Substrate. DNA and Cell Biol 32(3): 90-97. doi: 10.1089/dna.2012.1902.

Liu SC, Tang J, Zhang XH, Gao YY, Ma F, Yang QY (2012) Study on the interaction of Prodigiosin with bovine serum albumin by spectroscopic methods. Spectroscopy 27: 19-26. doi: 10.3233/SPE-2012-0561.

Liu X, Wang Y, Sun S, Zhu C, Xu W, Park Y, Zhou H (2013) Mutant breeding of *Serratia maracescens* strain for enhancing prodigiosin production and application to textiles. Prepar Biochem Biotechnol 43:271-284. doi: 10.1080/10826068.2012.721850. Bioorg Med Chem 24(7): 1504-12. doi: 10.1016/j.bmc.2016.02.020.

San-Lang W, Nguyen VB, Doan CT, Tran TN, Nguyen MT, Nguyen AD (2020) Production and Potential Applications of Bioconversion of Chitin and Protein-Containing Fishery Byproducts into Prodigiosin: A Review. Molecules 12: 2744. doi: 10.3390/molecules25122744.
